# Secured Secret Sharing of QR Codes Based on Nonnegative Matrix Factorization and Regularized Super Resolution Convolutional Neural Network

**DOI:** 10.3390/s22082959

**Published:** 2022-04-12

**Authors:** Ramesh Velumani, Hariharasitaraman Sudalaimuthu, Gaurav Choudhary, Srinivasan Bama, Maranthiran Victor Jose, Nicola Dragoni

**Affiliations:** 1 Institute of Electrical and Electronics Engineers (IEEE), Aruppukottai 626101, India; velumaniramesh@ieee.org; 2 School of Computing Science and Engineering (SCSE), VIT Bhopal University, Bhopal 466114, India; hariharasitaraman@gmail.com; 3 DTU Compute, Technical University of Denmark (DTU), 2800 Lyngby, Denmark; gauch@dtu.dk; 4 Kalasalingam Academy of Research and Education Krishnankovil, Srivilliputtur 626128, India; haribama111@gmail.com; 5 Noorul Islam Centre for Higher Education Kumaracoil, Thucklay, Kanyakumari 673012, India; mvictorjose@yahoo.com

**Keywords:** secret sharing, quick response code, Nonnegative Matrix Factorization, super resolution, convolutional neural network, structural regularization, basis matrix, coefficient matrix

## Abstract

Advances in information technology have harnessed the application of Quick Response (QR) codes in day-to-day activities, simplifying information exchange. QR codes are witnessed almost everywhere, on consumables, newspapers, information bulletins, etc. The simplicity of QR code creation and ease of scanning with free software have tremendously influenced their wide usage, and since QR codes place information on an object they are a tool for the IoT. Many healthcare IoT applications are deployed with QR codes for data-labeling and quick transfer of clinical data for rapid diagnosis. However, these codes can be duplicated and tampered with easily, attributed to open- source QR code generators and scanners. This paper presents a novel *(n*,*n)* secret-sharing scheme based on Nonnegative Matrix Factorization (NMF) for secured transfer of QR codes as multiple shares and their reconstruction with a regularized Super Resolution Convolutional Neural Network (SRCNN). This scheme is an alternative to the existing polynomial and visual cryptography-based schemes, exploiting NMF in part-based data representation and structural regularized SRCNN to capture the structural elements of the QR code in the super-resolved image. The experimental results and theoretical analyses show that the proposed method is a potential solution for secured exchange of QR codes with different error correction levels. The security of the proposed approach is evaluated with the difficulty in launching security attacks to recover and decode the secret QR code. The experimental results show that an adversary must try 2^58^ additional combinations of shares and perform 3 × 2^88^ additional computations, compared to a representative approach, to compromise the proposed system.

## 1. Introduction

Modern commercial applications employ QR codes in brand promotion, enriching consumer usage experience, interactive labeling for sharing product information, including promotional videos, web links, etc. In addition, QR codes are integrated with service platforms of governments for the effective delivery of utilization and administrative services to the public. The simplicity of QR code generation and scanning with cheap smart phones and IoT has harnessed their extensive adaptation by commercial and nonprofit organizations [1]. For an example, QR codes allow the consumer to connect to the IoT with a simple smartphone or tablet scan. Having all objects marked with a QR code or barcode means improving the retail environment for consumers because they will be more educated about the item before purchasing, and they will be able to check for an item’s availability. On the other hand, they are also susceptible to tampering and duplications for illegal financial benefits and counterfeiting authentic goods [2]. Security investigations have reported huge losses to commercial organizations that are ascribed to the flooding of fake goods carrying authentic QR codes. Several mechanisms have been proposed so far for protecting the QR codes against attacks.

**Figure 1:**
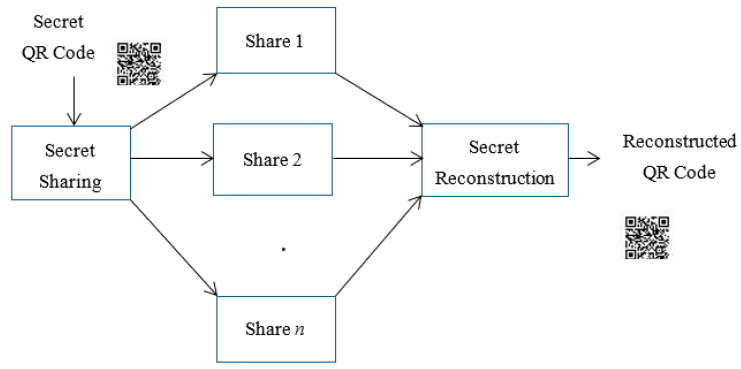
*(n*,*n)* QR code sharing.

The primitive QR code security approaches embedded the QR codes within cover images in the spatial or frequency domain [3,4]. Later, watermarks were embedded in the frequency domain of the codes employing standard image transformations [5,6] such as Discrete Wavelet Transform (DWT), Discrete Cosine Transform (DCT) and Discrete Fourier Transform (DFT). Later, spatial domain watermarking schemes exploiting the structure and error correction [7,8] capabilities of the QR codes were proposed. In these schemes, the QR codes with distortions were not readable and required additional morphological and interpolation operations to be recovered for reading. Further, if the error correction level was high and the embedded data was not encrypted, the QR code and the hidden data could be read by the attacker. In a similar method proposed by Chen [9], the QR code was embedded with message authentication code and cryptographic signature, exploiting the redundancy of error correction codes. Scanned with a conventional barcode scanner, this stego QR code revealed only the public information. The authentication data embedded within the code could be retrieved only if the barcode structure and embedding procedure was known. On successful extraction, the authenticity of the QR was verified. Of late, secret-sharing schemes are widely used in securing the QR codes from malicious attacks. These approaches share the QR code as secret shares among participants and recover the QR code from the shares. With these schemes, better robustness can be achieved. The schematic of the *(n*,*n)* secret-sharing scheme for sharing a QR code as a secret is shown in Figure 1.

The effectiveness of QR codes in healthcare applications has been demonstrated by various researchers recently [10,11]. Earlier, Feng [12] and co-workers demonstrated the fabrication of an immune-chromatographic assay labeled with QR codes for rapid biomedical diagnosis with Google Glass. In this approach, a QR code generator creates a QR code identifier for one or more diagnostic tests. Attaching a QR code label facilitates the automatic identification of the test of interest and other relevant data such as the patient details. Jamu [13] and co-workers evaluated the feasibility of utilizing the QR codes in capturing the real-time clinical data in an inpatient clinical environment and reported their effectiveness. A QR code-based diagnostic assay for detection and tracking of malaria has been proposed by Mthembu [14] et al. The QR codes signifying positive, negative and invalid test results integrated with diagnostic kits facilitate the immediate acquisition of clinical data from the point of study to the central laboratories, with the aid of Google Analytics. This approach is found to be effective in the surveillance investigations of diseases. In addition, many researchers have started exploring the integration of QR codes in various clinical applications.

In this intriguing context, this paper proposes a novel approach for sharing QR codes based on NMF [15] and SRCNN [16]. Particularly, we introduce variants of these classical approaches called the multi-layer NMF and structure regularized SRCNN in realizing the proposed system. The inherent characteristic of the NMF in creating component matrices with nonnegative elements is exploited in this work, in the creation of secret shares from QR codes at the sender side. These shares are combined to reconstruct the QR codes at the other end. The proposed scheme is featured as an *(n*,*n)* secret-sharing approach, in which all the shares are essential for reconstruction of the secret QR code. The structure regularization constraint ensures that the structural elements of the reconstructed QR code are intact. This scheme is ideal for sharing secret data as QR codes, to establish trust among a group of participants, creating a secured environment.

The contributions of this research are as below.

1. This paper proposes a novel secret-sharing mechanism for sharing QR codes as basis and coefficient matrices constructed by a multi-layer NMF as secret shares.
2. The QR codes are recovered by computationally less expensive Nonnegative Matrix Reconstruction operations and structure regularized SRCNN on the secrets.
3. The proposed approach eliminates the need for an explicit carrier image to embed the secret shares, as the individual shares do not carry significant information for an attacker.
4. This approach is free from pixel-expansion problems as the shares are not embedded for sharing.
5. The security of the secret can be improved by increasing the number stages of NMF for decomposition of the shares.
6. This approach is suitable for QR codes with different error correction levels as the secret-sharing and reconstruction operations are the same for all sizes of secrets.

Pixel expansion problems encountered in conventional secret-sharing schemes are completely averted in the proposed scheme, as NMF generates the shares by factorization. The SRCNN is applied to recover the QR code from the approximate version obtained from the secret shares. Experimental results with a standard dataset show that the proposed system is an eventual solution towards the realization of anti-counterfeit QR codes. This paper is organized with a review on conventional secret-sharing schemes and QR code-based secret-sharing schemes in Section 2. The mathematical foundations of the proposed system are described in Section 3 and the architecture of the proposed system is given in Section 4. The experimental results, comparative analyses, security analyses and interpretations are given in Section 5. The paper is concluded in Section 6.

## 2. Literature Review

Secret Image Sharing (SIS) schemes share a secret image as a number of secret shares or shadow images among the participants and recover the secret image, combining sufficient number of shares. Visual Secret Sharing (VSS) and Polynomial-based Secret Sharing (PSS) are the popular SIS approaches. VSS schemes reconstruct the secret image simply stacking the secret shares. These schemes based on the logical XOR operations are characterized by lossy recovery and low visual quality of reconstructed secret images [17]. In the earliest (*k*,*n)* PSS scheme proposed by Naor and Shamir [18], the secret image is divided into *n* shares, where at least *k* out of *n* shares are required for secret image reconstruction. Though this scheme is secure, it is characterized by storage overheads, as each shadow image is of the size of the secret image. A variant of this scheme proposed by Thien and Lin [19] reduced the size of each shadow image by 1/*k* of the secret image. However, in this method, traces of the secret image are evidenced in the shares and the secret can be reconstructed from insufficient number of shares, forsaking security. Though lossless recovery of secret image is achieved by this method, it suffers from random pixel expansion. Further, other PSS schemes have also been proposed featuring lossless recovery. The scheme proposed by Yang et al. [20] based on polynomials in the Galois Field, exhibits higher computational costs compared to other methods. Similarly, a lossless scheme proposed by Ding et al. [21] also suffers from limitations such as random shape changes, large shadow size and high computational complexity. In a *(k*,*n)* PSS scheme proposed by Zhou et al. [22], the shadow size is reduced to 1/*k* − 1 of the secret image. This method embeds the first *k* − 1 coefficients to reduce the shadow size. A *(n*,*n)* visual secret-sharing scheme based on XOR operations is proposed in [23], which shares the secret as *n* meaningful shares among *n* participants. The authors of this paper claim that this method is superior to conventional methods as the drawbacks such as pixel expansion, alignment of shares for reconstruction, loss of contrast, need for an explicit codebook for construction, etc., are mitigated.

Though creation and recognition of QR codes are simple, incorporating them in the business workflow of enterprises poses severe security risks, as QR codes are vulnerable to copy–paste attacks. A QR code can allegedly be used as an attack vector for threatening the reputation of an organization. Sharing a QR code securely among a group of people as secret shares and recovering the QR code from the shares will be a potential solution to enforce trust among a group of people. The significant difference between sharing images and QR codes is that the QR codes must be decodable after recovery. This requirement imposes a stringent constraint on the implementation of the QR code secret-sharing schemes.

Several attack scenarios such as Cross-Site Request Forgery attack (CSRF) [24], Cross-Site Scripting (XSS) attack [25], social engineering, phishing and pharming attacks can be launched, making minimal changes to genuine QR codes. Various empirical studies on the use of QR code as an attack vector are demonstrated in [26]. In order to prevent these attacks, QR code-sharing schemes must avoid information leakage in the secret shares, making reconstruction difficult. In addition, limitations of conventional secret-sharing schemes such as pixel expansion, memory overheads and computational costs must also be reduced or overcome in these schemes. Further, readability requirements also make QR secret-sharing a challenging task. Hence, there are only very few works in this context, discussed in this section.

Lin [27] proposed an *(n*,*n)* secret-sharing scheme in which the secret is divided into *n* shadows. Each shadow along with the authentication code is embedded as a pair (s_i_,v_i_) into the data codewords of each cover QR code QR_i_. At the other end, (s_i_,v_i_) pair is extracted from each QR_i_ and all the shares are combined to reveal the secret. This scheme verifies the integrity of each share with the verification code, generated using a master key and a hashing function. A *(k*,*n)* secret-sharing scheme proposed in [28] shares a secret image as *n* QR code shares and provides two approaches for revealing the secret, one by stacking the QR code shares and the other by performing XOR operations. This approach called a VSS-based QR code (VSSQR) scheme, exploits the error correction capabilities of the QR codes to generate QR code shares to share images. The secret image can be revealed by stacking a sufficient number of QR-code shares in low-resource settings. Further, this method also facilitates lossless recovery of a secret image by XOR operations among the shares. In an *(n*,*n)* secret-sharing mechanism proposed in [29], a secret message is encoded as a secret QR code and shared among *n* participants as QR code shares. The secret message is decoded from the QR code revealed by combining these *n* shares. Similarly, a cooperative secret-sharing protocol proposed in [30], embeds secret messages within QR codes and distributes them to *n* participants such that each QR code carries both public and private information. Public information is readable by conventional QR scanners while the private message is extracted using a symmetric key. This message is then decoded with the private key of a participant to extract the share. These shares are combined to extract the secret messages.

Liu et al. [31] have proposed a (3,3) threshold secret-sharing scheme called the VSS-QR code. This approach encodes the binary QR codes into three color shares and recovers the QR code by stacking them. Yu et al. [32] present a three-level QR coding scheme, embedding sensitive information within a carrier QR code in three steps, revealing only the public information of the carrier at the first layer.

Recently, Huang et al. [33] presented an *(n*,*n)* threshold QR code secret-sharing scheme, exploiting the error-correction capability of QR codes to enhance the security of the code. In this approach, a secret message encoded as a QR code is shared as n shares among n participants, where all the shares have the same version and error correction level similar to the secret QR code. A codeword is associated with each secret share such that each codeword comprises a data codeword and error-correction codeword. The secret is revealed by applying XOR operation on the codewords. This approach successfully decodes the secret from the tampered codewords, exploiting their error correction ability.

In a similar scheme, VSS is extended to the security of web services by Chen et al. [34]. WeChat is one of the most popular messaging apps to send messages, pay bills, share photos and browse news. WeChat Mini-Programs allow developers to run web services, get feedback from users and even monetize their services. By scanning a WeChat Mini Program code, the corresponding program can be accessed. Security of these codes is a concern for users and developers. An *(n*,*n)* Mini Program Visual Secret-Sharing Scheme (MPVSS) proposed by the authors is used for identification and control of the program users. In this scheme, a secret program code is encoded into n shares using n cover codes. The secret code is decoded by XOR operation on the shares, exploiting the error-correction abilities of the code. A comparison of the representative VSS schemes is presented in Table 1.

Researchers have shown that nonnegativity is a useful constraint for matrix factorization to learn parts representation of the data. The nonnegative-basis vectors obtained by factorization are used in distributed and sparse combinations to improve expressiveness in reconstructions. NMF is a classical mathematical tool employed in various domains to analyze data from different perspectives. Due to its demonstrated flexibility in the design of scalable and efficient approaches for solving large-scale problems and accuracy of solutions for real-world problems with noisy data, the Frobenius [35] norm-based NMF is widely applied as in [36].

Effectiveness of NMF in capturing the intrinsic geometric properties of images in image-classification problems was demonstrated by Cai and Sun [37]. Shan et al. [38] employed rank adaptive NMF in handwritten character recognition to extract local features of images. In combination with the Extreme Learning Machine (ELM) and k-Nearest Neighbor (KNN) algorithms, NMF was found to significantly reduce the image dimensions and improve classification accuracies. Symmetric Sparse NonNegative Matrix Factorization (ssNMF), a variant of NMF proposed by Li et al. [39], in combination with sparse coding was demonstrated to be effective in the detection of community structure of the brain from magnetic resonance images.

The effectiveness of NMF in digital-content security applications has also been demonstrated in various research papers. The earlier works in this context are digital watermarking schemes for audio, image and video data. In these schemes, NMF is combined with other mathematical transformations such as Singular Value Decomposition (SVD), DFT, DWT, etc., to physically embed a watermark. In a VSS scheme proposed by Wang [40] based on Discrete Fractional Fourier Transform (DFRFT) and NMF, the master and secret shares are constructed by applying NMF on the secret image. Similarly, a secret-sharing scheme for sharing Chinese characters represented as binary images was proposed in [41]. In this work, the authors employed a modified NMF in which the elements of *W* and *H* were closer to 1 or 0. Though this paper claims that the Chinese characters could be shared as multiple parts, experimental results were shown for 1-stage NMF only. Further, no quality metrics were reported in this paper. However, similar works were not reported so far in this context.

An image-hashing approach proposed by Karsh et al. [42], employing Projected Gradient Nonnegative Matrix Factorization (PG-NMF) for capturing local features of an image was demonstrated to effectively localize the counterfeit area in an attacked image. In an image encryption and multiplexing system proposed by Chang et al. [43], NMF and digital holography were employed in the secured exchange of keys for protecting the digital images. In this method, NMF was applied on noise-like digital holograms generated out of the candidate image, resulting in basis and weighted image matrices. The basis images were secured as encrypted data while the column vectors in the weighting matrix served as the keys distributed among participants. In a digital watermarking scheme proposed by Chen [44] et al., generalized NMF that does not impose a dimension- matching constraint, was employed to embed an image within an image. In this approach, the host image was factored into a basis matrix *A* and a coefficient matrix *B*. Though the authors claimed that the dimension of *A* was (1,*n*)*,* which reduces the number of basis components, it was found that each element in the row vector *A* was a 2-dimensional representation of the original host image. Watermark embedding was performed by directly replacing the smallest image component of *A*. This scheme resulted in severe pixel expansion.

Loss of resolution in reconstructed images is a major drawback of visual cryptographic schemes, as discussed in the work of Weir and Yan [45]. Effectiveness of super resolution algorithms in the construction of High-Resolution (HR) images from Low-Resolution (LR) images in pan-sharpening of aerial images, medical image analysis for minimum invasive robotic surgery, sign and number plate reading, iris recognition, etc., is demonstrated in the literature. Loss of quality in a reconstructed image was attributed to pixel expansion rate and relative difference in weights of the shares generated from different color levels as discussed in the work of Wu et al. [46]. Loss of resolution of a reconstructed image can be reduced by minimizing pixel expansion and maximizing the relative difference between the weights of the shares. However, this issue can be resolved by improving the resolution of the recovered images with single-image, super-resolution algorithms. These algorithms are broadly classified as statistical, prediction-based, edge and patch or example-based methods.

A thorough investigation of these methods by Yang et al. [47] showed that example-based methods reported in [48,49] achieve state-of-the-art performance. Conventional example-based methods exploit the internal similarities of a given image to perform a mapping between LR images and relevant HR images in a dataset for construction of HR images. Sparse coding is a kind of example-based, super-resolution method, which employs dictionaries in the construction of HR images. In this method, initially, overlapping patches densely cropped from the LR image are encoded into an intermediate sparse representation using an LR image dictionary. HR images are reconstructed from HR image patches estimated from HR dictionaries, using sparse coefficients. This method involves optimization of learning operations from dictionaries and mapping functions, which is realized using the SRCNN, which optimizes the learning, mapping and patch aggregation operations.

Our extensive review reveals that QR codes are employed mostly as carriers in QR code-based secret-sharing schemes. Further, the structure of the cover QR codes and the nature of error correction mechanisms play a vital role in determining the embedding capacity of the cover QR codes, which requires lengthy computations. It is also evident that NMF-based VSS schemes have not been explored extensively. In pursuit of new secret-sharing approaches, the proposed work intends to exploit the property of NMF in representation of image parts for creating secret shares from a QR code and recovering it from the shares. Further SRCNNs are demonstrated to effectively capture the relationship between LR and HR patches.

## 3. Materials and Methods

This section may be divided by subheadings. It should provide a concise and precise description of the experimental results, their interpretation, as well as the experimental conclusions that can be drawn.

### 3.1. Dataset and Implementation Details

The proposed system is tested with the dataset accompanying [50], which contains 34 QR codes with error-correction levels L, M, Q and H. The QR codes are of dimensions 29 × 29, 33 × 33, 41 × 41, 45 × 45, 53 × 53, 57 × 57, 61 × 61 and 77 × 77 in PNG format. Demonstrating the ability of the proposed QR code-sharing approach to reconstruct the QR codes of varied sizes from the secrets shared is essential to demonstrate the robustness of the system. This evaluation is required to prove the flexibility, reliability and generalization ability of the secret-sharing scheme. Further, it also facilitates the identification of prospective applications of the system based on the requirements of applications such as medicine, science, engineering and finance. The size of the QR codes is a major concern in realizing security mechanisms such as privacy, data integrity and authentication. This dataset has QR codes of sufficiently varying sizes with different error-correction levels to test the system.

The secret-share construction and secret-reconstruction processes of the proposed system are implemented with Matlab2020b software. The Zxing [51] library is used for decoding the reconstructed QR codes super-resolved with the structure regularized SRCNN.

### 3.2. Multi-Layer Nonnegative Matrix Factorization

**Figure 2:**
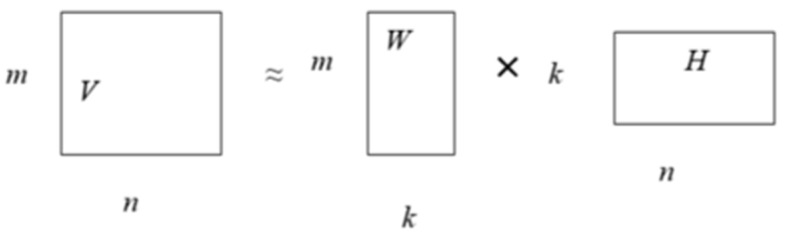
NMF reconstruction.

NMF is a class of techniques for approximately factorizing a matrix *V* of size mxn into two nonnegative matrices *W* and *H,* each of size m × k and k × n as shown in Equation (1), where *W* is the basis matrix and *H* is the coefficient matrix. In linear algebra, a basis vector is used to represent a concise and finite description of an infinite vector space. The reconstruction of *V* is shown in Figure 2.

(1) V≈WH

The factorization of *V* into *W* and *H* is not unique, as different values of *k* yield different *W* and *H* matrices. It has been shown empirically that for any matrix *V*, better approximation of *V* is achieved when the condition is satisfied as in the expression (2).

(2) k≤min(m,n)

The smallest value of *k*, resulting in *V* = *WH* is called the nonnegative rank of *V*, expressed as *rank*_+_(*V*) as in (3).

(3) $$rank(V)\leq rank_{+}(V)\leq \min(m,n)$$

Based on the cost functions used in the divergence measure between *V* and *WH*, there exist variants of *NMF*. The squared-error version of NMF employs iterative update rules for minimizing the divergence as given in Equation (4).

(4) $$F(W,H)={\left\|V-WH\right\|}_{F}^{2}$$

where *F* is called the Frobenius norm.

**Figure 3:**
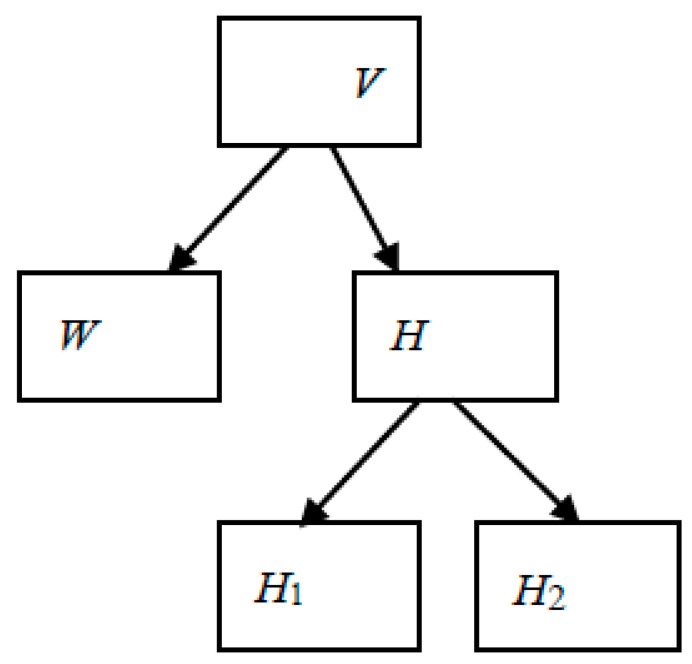
2-stage NMF.

This paper proposes a QR code secret-sharing scheme realized with a two-layer NMF model as shown in Figure 3. This model is based on the multi-layer NMF [52], mathematically represented as in Equation (5).

(5) $$V\approx WH_{1}H_{2}\ldots H_{n}$$

where

[W,H]=NMF(V)

$$\begin{array}{l} \left[H_{1},H_{2}]=NMF(H\right)\\ \left[H_{3},H_{4}]=NMF(H_{2}\right)\\ \left[H_{5},H_{6}]=NMF(H_{4}\right) \end{array}$$

…

$$\left[H_{n-1},H_{n}]=NMF(H_{n-2}\right)$$

From the above it can be seen that, in every stage of decomposition, the basis components are preserved and the coefficient matrices are factorized. By generalization, the mathematical model of the 2-stage *NMF* is given in Equation (6).

(6) $$V\approx WH_{1}H_{2}$$

### 3.3. SRCNN with Structural Regularization

**Figure 4:**
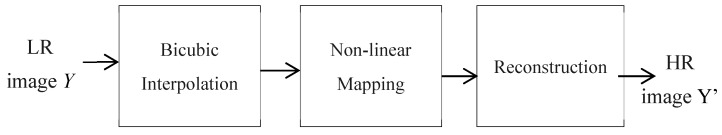
SRCNN architecture.

The SRCNN features a simple convolutional neural network structure, which directly learns an end-to-end mapping between LR and HR images, without the need of any pre- and post-processing operations. Given a ground-truth image *X* and its LR representation Y, the SRCNN constructs an HR image *Y*’ from *Y* such that it is equivalent to *X*. Image super resolution is performed by the SRCNN in three steps as below, illustrated with Figure 4.

**(1) Patch extraction and representation:** In this operation, patches called feature maps representing essential features of *Y*’ are created by convolving a filter with *Y* and are represented as a high-dimensional vector.

**(2) Nonlinear mapping:** This operation nonlinearly maps each feature map into a high-dimensional space.

In this step, a convolution filter introduces a high degree of non-linearity to achieve higher accuracy.

**(3) Reconstruction:** This operation aggregates the feature maps to generate the final HR image *Y*’, which is expected to be similar to the ground-truth image *X*.

In this research, we introduce a structural regularization constraint to ensure that the structural details captured from *Y* to *Y*’ are intact. The problem of reconstruction of *Y*’ from *Y* is formulated as in (7), where *D*, *S* and *N* refer to the down-sampling operator, blurring operator and the noise, respectively.

(7) $$Y=DSY^{’}+N$$

The super-resolved image *Y*’, which closely matches the ground truth *X*, is obtained by minimizing the mathematical model of (7) as in Equation (8). The first term in this equation is called the fidelity term, which penalizes the difference between the reconstructed image *Y*’ and the LR image *Y*. The second term is called the structural regularization term, where Rs is regularization factor and *λ_s_* is the weight factor that balances the trade-off between fidelity and structural similarity.

(8) $$X=\min_{Y}({\left\|DSY’-Y\right\|}^{2})+\lambda_{s}R_{s}$$

We enforce structural similarity between *Y* and *Y*’ by defining *R_s_* as a cross-gradient term in Equation (9), which aligns the gradients of the individual image patches in *y_i_* and *y_i_’* of the images *Y* and *Y*’.

(9) $$R(y_{i},y{’}_{i})=\frac{1}{2}\int_{\Omega }{\left|\nabla_{y_{i}}\nabla_{y{’}_{i}}\right|}^{2}dy$$

SRCNNs focus only on the details within a patch without considering the structural relationship between an image patch and neighboring regions. Introduction of the structural constraint ensures that the structural components from the LR image are preserved in the HR image.

## 4. Proposed System

The proposed work is implemented in three phases viz. secret-share construction, secret reconstruction and image super resolution, described in the following subsections with schematic diagrams and algorithms.

### 4.1. Secret-Share Construction

**Figure 5:**
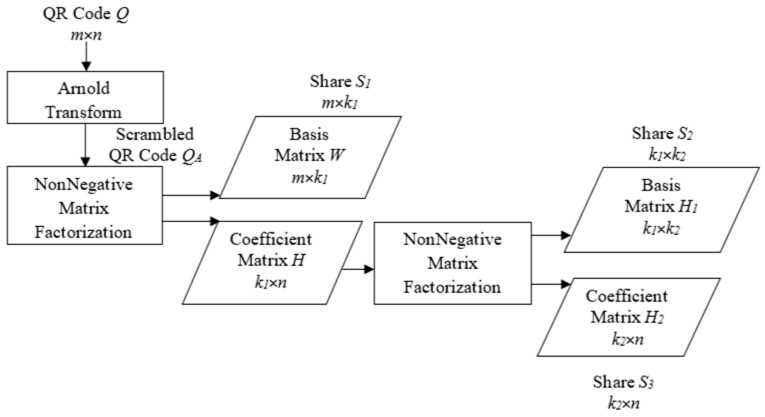
Secret-share construction.

Construction of secret shares from the QR code is illustrated with Figure 5. Initially, the QR code *Q* is scrambled with Arnold Transform to generate the scrambled QR code *Q*_*A*_ and the rank of the matrix *k*_1_ is determined. *Q*_*A*_ is then factored into basis matrix *W* and coefficient matrix *H* of dimensions *m* × *k*_1_ and *k*_1_ × *n*, respectively. The basis component *W* is secured as secret share *S*_1_. The rank *k*_2_ of *H* is determined and it is further factored in to *H*_1_ and *H*_2_ of dimensions *k*_1_ × *k*_2_ and *k*_2_ × *n*, respectively, which are secured as secret shares *S*_2_ and *S*_3_, respectively. The steps for implementation of this process are given as Algorithm 1.
**Algorithm 1. Secret Sharing**Input: QR code *Q*, number of iterations *i* Output: Secret shares *S*_1_, *S*_2_ & *S*_3_ *Method:*  1. Apply Arnold Transform on *Q*   *Q_A_*
*← Arnold Transform*(*Q,i*)  2. Find the rank of *Q_A_*   *k*_1_*←rank*(*Q_A_*)  3. *Factor Q_A_* into base and coefficient matrices   *[W,H]←NMF*(*Q_A_,k_1_*)  4. *Find the rank of H*   *k*_2_*←rank*(*H*)  5. *Factor H* into base and coefficient matrices   *[H*_1_, *H*_2_*]←NMF*(*Q_A_*,*k*_2_)  6. *Construct the Secret Shares*
     a. *S*_1_*←W*     b. *S*_2_*← H_1_*     c. *S*_3_*← H_2_*

### 4.2. Secret Reconstruction

**Figure 6:**
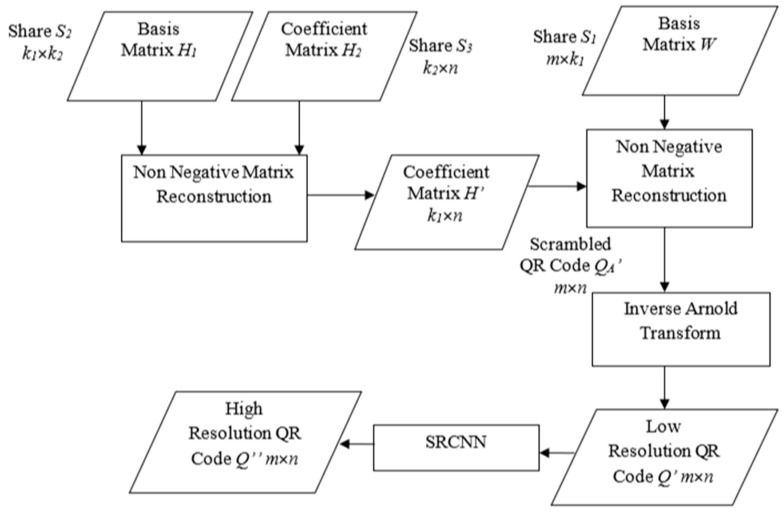
Secret-image reconstruction.

Extraction of the QR code from the secret shares is shown in Figure 6. Initially, the shares *S*_2_ and *S*_3_ are multiplied to generate *H*’, the approximation of *H*. The secret share *S*_1_ is multiplied with *H*’ to get the approximation *Q_A_*’, of the scrambled QR code *Q*. Inverse Arnold Transform is applied on *Q_A_*’ to retrieve the unscrambled QR code. The SRCNN is applied on the reconstructed QR code *Q*’ to generate a high-resolution QR code *Q*″. Algorithm 2 lists the steps for implementing this process.
**Algorithm 2. Secret Reconstruction**Input: Secret shares *S*_1_, *S*_2_ & *S*_3_, number of iterations *i* Output: Reconstructed secret *Q*″ Method:  1. Reconstruct the coefficient matrix *H*’   *H*’← *S*_2_* *S*_3_  2. Reconstruct the Arnold Transformed Secret *Q*’*_A_*   *Q*’*_A_*← *S*_1_* *H*’  3. Apply Inverse Arnold Transform on *Q*’*_A_*   *Q*’ *← Arnold Transform*(*Q*’*_A_,i*)  4. Reconstruct the secret by Super Resolution     a. *Q*″←*SRCNN(Q’)*

Experimental results have shown that image reconstruction from the *NMF* component matrices provides an approximation of the original image. Since the image is subjected to two levels of decomposition in the proposed system, the reconstructed image cannot provide a best approximation of the original image. The SRCNN-based reconstruction algorithm is implemented in this system to improve the quality and in turn the readability of the reconstructed QR code.

### 4.3. Image Super Resolution

**Figure 7:**
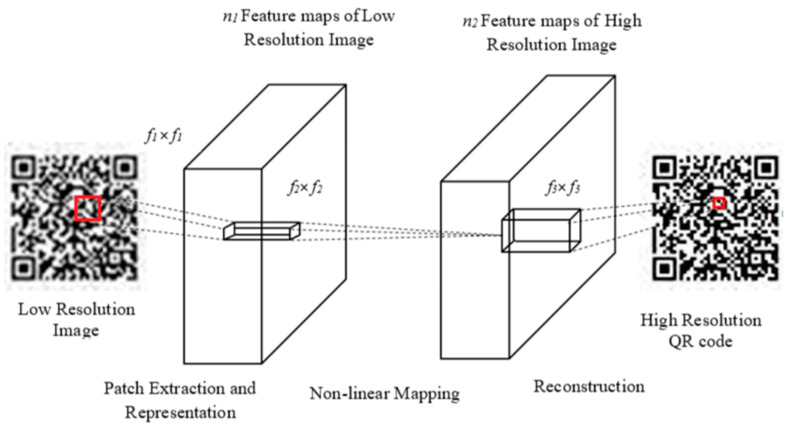
QR code super resolution.

The schematic of the SRCNN is shown in Figure 7 for reconstruction of an HR QR code from its LR version. In the proposed work, we employ the SRCNN constrained by structural regularization in recovering the readable QR code from the approximate version reconstructed by combining the secret shares constructed with *NMF*. Initially, the binary QR codes in the dataset are transformed to RGB to enhance the image resolution. The first stage of the SRCNN convolves the LR image with filters of size *f*_1_ × *f*_1_ for *n*_1_ times to represent each patch as an *n*_1_ dimensional vector. In the second stage, each *n*_1_ dimensional vector is transformed into an *n*_2_ dimensional vector by convolution with *n*_2_ filters of size *f*_2_ × *f*_2_. Each *n*_2_ dimensional vector is the HR representation of a patch in the LR image. Finally, these vectors are convolved with filters of size *f*_3_ × *f*_3_ to construct the super-resolved image.

## 5. Experimental Results and Discussions

The proposed system was run in an Intel i5 processor with NVIDIA GeForce 920 MX GPU on the QR codes in the dataset described in Section 3.1. The number of iterations i for Arnold transform is assumed to be 4, while rank values *k*_1_ and *k*_2_ depend on the input matrix. The 9-5-5 SRCNN model with hyper parameters *f*_1_ = 9, *f*_2_ = 5, *f*_3_ = 5, *n*_1_ = 32 and *n*_2_ = 64 is employed in the QR code reconstruction. For structural regularization, *λ_s_* is initialized to 0.2 after empirical evaluations. In Table 2, the QR codes, secret shares, and reconstructed QR codes along with image quality metrics are shown for 10 best samples in terms of SSIM values in decreasing order.

From the results in Table 2, it is seen that the reconstructed QR codes possess reasonable visual quality from the PSNR values. The SSIM values also signify the similarity between the original and reconstructed QR codes. However, readability of the QR code is the prime requirement compared to the visual quality and similarity metrics. Secret sharing is accomplished in the proposed system only if the reconstructed secret is decodable. It has been verified that all the QR codes with different error-correction levels are decodable by the Zxing decoder. Finally, the decoded QR codes are transformed as binary images for performance evaluation of the system as the original dataset contains binary QR codes.

It is seen that the highest SSIM value of 0.9373 is achieved for a QR code of size 29 × 29, while the highest PSNR of 32.3889 dB is attained on recovering a QR code of 53 × 53. Further, analysis of the smallest values of the metrics show that least PSNR and SSIM values of 30.4143 dB and 0.8669 are attained on reconstruction of a 61 × 61 QR code. This result shows that performance metrics are irrespective of the size of the code. However, ability of the system to reconstruct decodable QR codes for all the test samples demonstrates the reliability of the system.

### 5.1. Performance Analysis

The experimental results clearly show that NMF is a suitable tool for secret sharing and recovery. Compared to the conventional secret-sharing schemes, which involve complex mathematical operations for secret sharing and reconstruction, the proposed system is comparatively simpler as it involves only factorization and multiplication operations. Further, the proposed system is devoid of the pixel expansion, a major limitation of the conventional secret-sharing schemes. Detailed comparisons of the proposed system with the existing secret-sharing systems with respect to different attributes are summarized in Table 2. This comparison is an extension of the comparisons presented in [22], which is a similar work in this context.

It has been shown that the complexity of NMF is *O*(*kmn*) in [53] and earlier literature, where *k* is the rank of the matrix. It is seen from Table 3 that the proposed system exhibits lossless recovery similar to few existing methods. Here, the shadow size depends on rank of the matrix *k,* which is less than *min*(*m,n*) for an *mxn* matrix. Hence the share size is either the same as or less than that of the secret. Similar to [21], the complexity of the proposed system is proportional to the number of shares or participants. Hence the complexity of the system is *O*(*n*).

The summary of the running times of the existing and proposed methods is presented in Table 4. For the proposed system, the time for secret-share creation and reconstruction is the average values of the time taken for these operations on the 34 sample QR codes.

From Table 4, it is seen that the time taken by the proposed method is comparatively very low. The comparisons presented in Table 3 and Table 4 are meant to provide a summary of the performance metrics only, as the proposed scheme is completely distinct from others. The methods proposed in [17,18,20,21] were based on evaluation of polynomials during secret-share construction and solving linear equations to reconstruct each pixel. The proposed scheme involves factorization for secret sharing, and reconstruction of secret is based on multiplications of shares and convolution in the SRCNN. Hence, the proposed method exhibits lower computational times with respect to [17,18,20,21] for both secret-share creation and secret reconstruction.

The (*n*,*n*) method in [23] involved generation of basis matrices and random shares, and conversion of random shares to meaningful shares for construction of secret shares. Hence, the proposed system has lower computational time for secret creation. The decryption involves only XOR operations between shares in [23] and therefore it is lower than the proposed system. Further, *C_SRCNN_* the complexity of the *SRCNN* is given in Equation (10).

(10) $$c_{SRCNN}=O((f_{1}^{2}n_{1}+n_{1}f_{2}^{2}n_{2}+n_{3}f_{3}^{2})S_{HR})\;$$

where

*f_i_* is the filter size
*n_i_* is the number of filters
*S_HR_* is the size of the HR image

In the proposed system, the complexity of the construction of the HR QR code from its LR representation is analogous to Equation (10). This complexity can be considerably reduced by modifying the values of hyper parameters. Further, there are no security constraints with the SRCNN.

As stated earlier, there are not many works reported on the sharing of QR codes, and for one such method presented in [41] explicit results are not available. Unlike the conventional secret-sharing methods that focus on the visual quality of the secret, the proposed system has the rigorous requirement of the readability of the secret that is achieved with the proposed system.

### 5.2. Security Analysis

The security of the proposed system relies on the imperceptibility of secret shares, attributed to the NMF factorization. From the experimental results, it is seen that the secret shares do not contain any trace of the secret. Further, the size of the shares depends on the ranks *k*_1_ and *k*_2_ of the candidate matrices *Q_A_* and *H*, respectively. It has been highlighted in literature that NMF is not unique for a given matrix *V*, and determination of the rank *k* is an NP hard problem. To recover the QR code from the shares, an attacker needs the following:

- Secret Shares *S*_1_, *S*_2_ and *S*_3._
- Sequence of combinations of shares.
- Number of iterations for inverse Arnold Transform *i.*

The QR code can be recovered only on combination of the shares in a particular sequence, i.e., *S*_2_ and *S*_3_ must be combined to recover *H*’, which must be combined with *S*_1_. In addition, the number of iterations *i* for scrambling the QR code and applying Arnold Transform also governs the security of the QR code. Inverse Arnold Transform with an erroneous number of iterations cannot recover the QR code. The degree of freedom for choosing the number of iterations is very large, which makes the retrieval of QR code difficult. In the proposed system, the ranks *k*_1_ and *k_2_* are evaluated from the candidate matrices, by determining the number of linearly independent rows or columns larger than a tolerance, using the *rank()* function of MATLAB.

The information theoretical and computational security analysis of the proposed system is as below.

**Information** **Theoretic** **Security**

*Any (n,n) secret-sharing scheme is information theoretic secure, if the secret cannot be revealed by any (n − 1) number of shares.*

In the proposed system, the shares have no visible components of the secret. According to the principle of *NMF*, the original matrix can be reconstructed only from the basis and coefficient matrices. In the proposed system, the secret shares are derived from the basis and coefficient matrices of a QR code, without which the QR code cannot be constructed. Hence, the proposed system is information theoretic secure.

**Computational** **Security**

*Any (n,n) secret-sharing scheme is computationally secure, if it is infeasible to invert the scheme from (n − 1) number of shares*.

It is very much evident that all the shares *S*_1*,*_ *S*_2_ and *S*_3_ must be combined for the reconstruction of *Q*’. Hence, it is infeasible to recover the secret unless all the shares are available. Generally, inversion of the secret-sharing scheme is associated with hardness assumptions of the computational procedures involved, such as use of encryption algorithms such as Advanced Encryption Standard (AES) and Cipher Block Chaining (CBC).

In the proposed system, the reconstruction involves only multiplication operations and inverse Arnold Transform. With either one or two shares available out of the three shares, it is not possible to construct the other shares and construct the secret, as each share is incrementally constructed starting from the factorization of the secret. Further, the security of the proposed system is demonstrated in this section with a quantitative analysis on the difficulty of brute force attacks and construction of imperceptible secrets from tampered shares.

#### 5.2.1. Brute Force Attack

Here, for a given *m*×*m* secret, we evaluate the number of computations required to generate the shares and reconstruct the secret. We arrive at the mathematical expressions for various computations and evaluate them with respect to the dataset used in our experiments. Similarly, we calculate the number of combinations required for brute-force attack on the dataset by the approach proposed in [23] and present a comparison.

In the proposed system, the secret shares *S*_1,_ *S*_2_ and *S*_3_ are of the dimensions *m*×*k*_1_, *k*_1_×*k*_2_ and *k*_2_×*n,* respectively. For the entire data set *m*=*n* and hence the share dimensions are *m*×*k*_1_, *k*_1_×*k*_2_ and *k*_2_×*m,* respectively for *S*_1,_ *S*_2_ and *S*_3._

The attacker needs to construct the individual shares by brute-force approach to recover the secret. Representing each pixel by either 0 or 1*,* the number of combinations for recovering the shares *S*_1,_ *S*_2_ and *S*_3_ is given in Equation (11). Further, *k*_1_ and *k*_2_ can assume any value from 1 to *m* according to Equation (2). This increases the number of combinations, and *C* can be expressed as in Equation (12).

(11) $$C=2^{mk_{1}+k_{1}k_{2}+k_{2}m}$$

(12) $$C=2^{mk_{1}+k_{1}k_{2}+k_{2}m}2^{m}2^{m}$$

Substituting *k*_1_ and *k*_2_ by the minimum value 1 in Equation (12), the minimum number of combinations is expressed as *C_min_* in Equation (13).

(13) $$C_{\min}=2^{m+1+m}2^{1}2^{1}=2^{2m+3}$$

Similarly, substituting *k_1_* and *k_2_* by the maximum value *m* in Equation (12)*,* the maximum number of combinations is expressed as *C_max_* as in Equation (14).

(14) $$C_{\max}=2^{m^{2}+m^{2}+m^{2}}2^{m}2^{m}=2^{3m^{2}+2m}\;$$

In the *(n,n)* secret-sharing scheme proposed in [23], 2*^m^*×*^m^*×*^n^* combinations are required to construct *n* shares, each of size *m*×*m.* A comparison of the number of combinations for constructing the shares in [23] and the proposed system is given in Table 4, for the secrets of varying sizes in our dataset.

**Figure 8:**
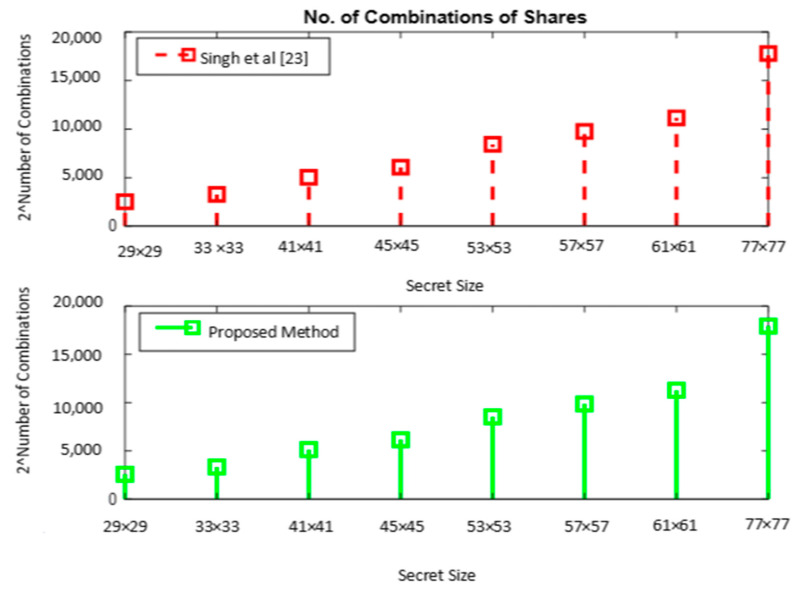
Brute-force attack—number of combinations of shares.

It is seen from Table 5 that the number of combinations for constructing 3 shares is very high for the proposed system compared to that of [23]. A graphical illustration of the above table in Figure 8 shows an identical pattern of the plots, which reveals that the number of combinations of shares linearly increases with the size of the secret. For a secret of size 29 × 29, the proposed method requires 2^58^ additional combinations to be tried compared to [23], which affirms the security of the proposed approach.

Further, the secret image is reconstructed by multiplying *S*_2_ and *S*_3_ first and the resultant with *S*_1_ in the proposed system*_._* As the attacker is unaware of the share labels, all the possible combinations must be tried. For 3 shares, the number of combinations is 2^3^. Two multiplication operations are required between the shares to recover the secret. From the above, the minimum and maximum number of total computations *T_min_* and *T_max_* can be expressed as in Equations (15) and (16).

(15) $$T_{\min}=2^{m+1+m}2^{1}2^{1}2^{3}2=2^{2m+7}$$

(16) $$T_{\max}=2^{m^{2}+m^{2}+m^{2}}2^{m}2^{m}2^{3}2=2^{3m^{2}+2m+4}$$

Further, inverse Arnold Transform must be applied on the scrambled secret reconstructed from the shares. Generally, an image of 2*^d^* pixels requires 3(2*^d^*^−2^)transformations to return to its original position. The number of transformations required for the test data set is given in Table 6. This further increases the number of computations to a greater extent.

Based on Equations (15) and (16) and column 3 of Table 6, the number of computations to be performed to recover the unscrambled secret is evaluated and given in Table 7. For a comparative analysis, this value is presented for [23], considering the XOR operations between *n* shares. For 3 shares, 2 XOR operations are to be performed which results in $2^{3m^{2}+1}$ computations.

**Figure 9:**
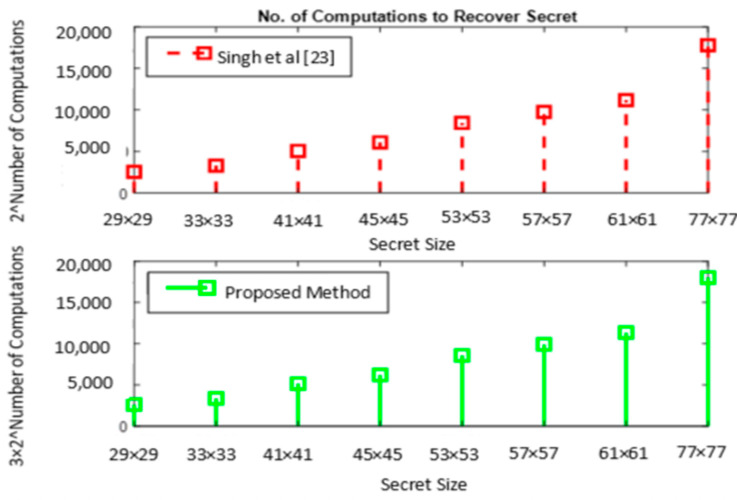
Brute-force attack—number of computations to recover secret.

A graphical representation of Table 7 shown in Figure 9 matches that of Figure 8, signifying the consistent behavior of the proposed model compared to [23] with respect to the number of combination of shares to be considered and the number of computations to recover the secret by bruteforce attack. Observation of the number of computations for a 29 × 29 QR code shows that 3 × 288 additional computations are required to recover the secret compared to [23]. However, it is seen that the number of computations required by the proposed system is very high compared to [23] as the secret size increases.

Finally, we present the number of computations evaluated with Equation (10) for the generation of HR secret from the LR secret in Table 8.

**Figure 10:**
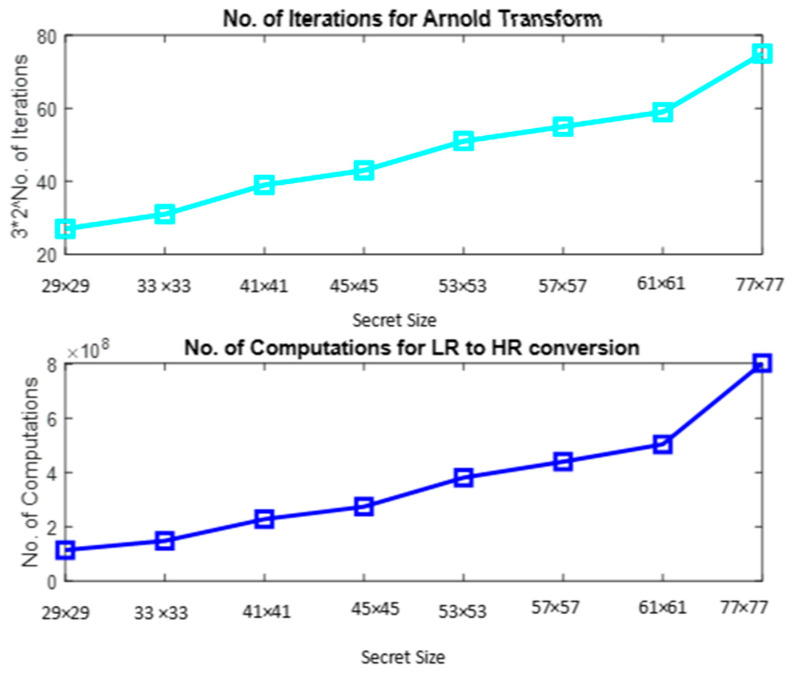
Brute-force attack—number of iterations for Arnold Transform and number of computations of Image Super Resolution.

Graphical illustrations of Table 6 and Table 8 are given in Figure 10, which summarizes the number of iterations of Arnold Transform and the number of computations required for super resolution of the secret obtained by brute-force attack. It is seen that the plots are identical, reinstating a linear increase in computational complexities with respect to the size of the secret.

It is seen that the number of computations increases with the size of the secret. From the number of computations required to construct a secret, it is seen that it is difficult for an attacker to construct a secret even with extreme computational resources.

#### 5.2.2. Attack on Shares

In secret-sharing schemes, the secrets are susceptible to intentional or accidental attacks. These shares may be tampered with by noise addition to the entire secret or selective modification of content. We show that the proposed system is resistant to these attacks with three experiments.

**Figure 11:**
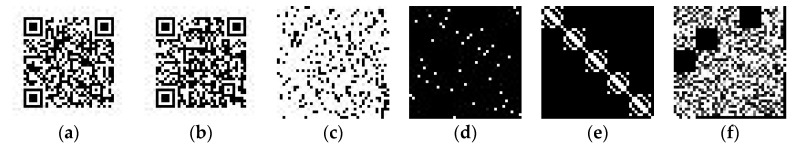
QR code reconstruction from tampered share (**a**) 5th QR code, (**b**) 6th QR code, (**c**) *S*_1_ (5th QR code), (**d**) *S*_2_ (5th QR code), (**e**) *S*_3_ (6th QR code), (**f**) reconstructed QR code.

The first attack is posed by completely replacing a secret share by other. It is seen from Table 2 that the secret share *S*_3_ exhibits a similar geometric pattern with significant values along the diagonals. This leads to an implication that *S*_3_ can be guessed and constructed with arbitrary significant values, challenging the security of the system. We experimented with this with the 5th and the 6th QR codes in [50], named *cite_09_Q_small* and *cite_10_Q_small*, respectively, each with dimension 41 × 41. All the shares generated from these QR codes have the same dimension. We have applied the reconstruction procedure on *S*_2_ of the 5th QR code and *S*_3_ of the 6th QR code to generate *H*’. The QR code *Q*’ is reconstructed by combining this *H*’ and *S*_1_ of the 5th QR code. The results of this experiment are shown in Figure 11. It is seen that the reconstructed QR code is completely indiscernible, testifying to the security of the proposed system.

**Figure 12:**
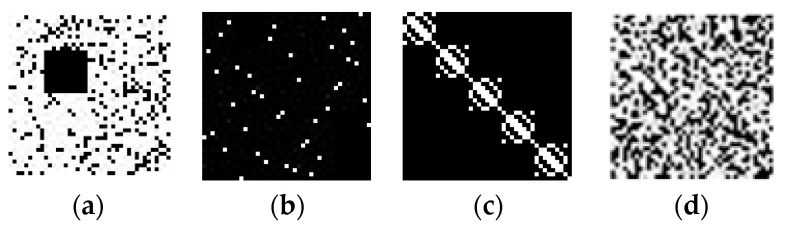
QR code reconstruction from selectively tampered share (**a**) *S*_1_ (6th QR code), (**b**) *S*_2_ (6th QR code), (**c**) *S*_3_ (6th QR code), (**d**) reconstructed QR code.

The second attack is launched by selective tampering of pixels in *S*_1_. The impact of this attack is tested by flipping some of the pixels in *S*_1_ of the 6th QR code and assigning 0 a block of 10 × 10 pixels. The shares corresponding to this QR code and the reconstructed secret are shown in Figure 12. It is seen that the reconstructed QR code is completely distorted.

**Figure 13:**
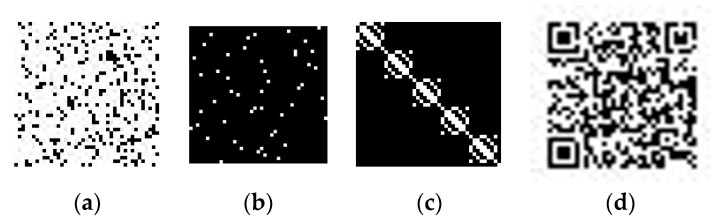
QR code reconstruction from share added with noise (**a**) *S*_1_ (6th QR code), (**b**) *S*_2_ (6th QR code), (**c**) *S*_3_ (6th QR code), (**d**) Reconstructed QR code.

The third attack is performed by addition of noise to *S*_2._ Salt-and-pepper noise of density 0.02 is added to the share *S*_2_ of the 6th QR code to study the effect of accidental noise addition. The shares and the reconstructed secret are shown in Figure 13. In spite of the secret being distorted, the structure of the secret is not completely lost. However, this secret is not decodable by the QR code reader. A noise density of 0.02 affects 2% of pixels in an image. We see that modification of a share by 2% introduces obvious distortions in the secret, rendering it unreadable.

### 5.3. Limitations and Future Works

Initially, we present the limitation of this research. This paper reports complete reconstruction and successful decoding of the QR codes in the dataset and also presents image-quality measures. However, most recent representative works do not contain such explicit results for comparison. The authors report complete recovery and decoding of a 41 × 41 QR code in [31] without obvious performance analysis of the (3,3) secret-sharing approach on a complete dataset. Similarly, in [32] only subjective results are presented for a small set of QR codes without objective results. Lack of comparative analysis with a standard dataset and quantitative measures restricts further explorations regarding the enhancement of the approaches. Further, due to lack of relevant works, security of the proposed system is compared only with that of [23], which is an (*n*,*n*) secret-sharing scheme. However, it shares the secret as meaningful shares.

From statistical evaluations and experiments by intentional modification of shares, it is seen that the proposed system is secure against brute-force and tampering attacks. However, appearance of uniform geometric patterns in all *S*_3_ is a vital security concern, which requires further investigation. The security of the system can be further improved by scrambling the shares and constructing meaningful shares, which do not raise suspicion. The *rank()* function employed in secret creation evaluates the rank of the matrix as the number of singular values of a matrix, greater than a default tolerance. Since NMF is applied in two stages in this work, the tolerance values can also be used to enforce the security by assuming suitable values. Further, the number of NMF stages can be increased to improve the security of the system.

Of late, color QR codes that have high data capacity and flexibility of encoding are widely used in product and service marketing. However, decoding them is a challenging task due to variations in color maps, channel interferences and the resolution of the camera. Further, applying VSS on color QR codes incurs additional costs, which increases the complexity of the system. The proposed VSS scheme can be extended to color QR codes with the same framework, using Nonnegative Tensor Factorization (NTF) in place of *NMF*.

## 6. Conclusions

While the existing secret-sharing systems employ QR codes as cover images to carry secret data, we envision the need for sharing QR codes as secrets. Recent medical IoT applications that transform real-time clinical data into QR codes require the protection of QR codes from unauthorized access and tampering. This security requirement can be realized with the proposed system, as demonstrated by our experimental works. This paper presents a novel QR code-sharing scheme exploiting the potential of *NMF* in part-based representation of images. It also harnesses the potential of SRCNN in the reconstruction of QR codes, which has not been attempted so far. With a standard dataset containing QR codes of varying error-correction levels, we have clearly demonstrated the efficacy of our system with experimental results, theoretic analyses and empirical evaluations of the security attacks. Though the first of its kind, this system imbibes the desirable features of a cryptographic system for secured exchange of sensitive data among participants. This research can be extended by customizing the proposed approach to diverse image classes such as micro QR codes, finger-prints and multi-modal digital images. Recently, color QR codes were introduced that can carry a relatively large amount of information. Though these QR codes feature high data density, they are prone to several problems such as color coding, detection, deblurring, etc., in reconstruction and decoding. The proposed system can be extended to share color QR codes enforcing additional constraints with the NMF and the SRCNN.

## Figures and Tables

**Table 1:** Comparative analysis of visual secret-sharing schemes.

Reference	Method Employed	Pros	Cons
Naor & Shamir [18] (1994)	Polynomial	Secrets are converted into unconditionally secure shadow image	Requires additional storage as each shadow image is of the size of the secret image
Thien & Lin [19] (lossy) (2002)	Polynomial	Size of shadow image is smaller than secret image	Traces of secret image are visible in the shadow images Method suffers from random pixel expansion
Yang et al. [20] (2007)	Polynomials in the Galois Field	Lossless recovery without pixel expansion	High Computational Cost
Ding et al. [21] (2018)	Polynomial scheme and modular algebraic recovery	Fully lossless recovery	Random shape changes Large shadow size High computational complexity
Zhou et al. [22] (2018)	Polynomial sharing and generalized Arnold permutation	Two adjacent pixels are used as secrets Leakage of secret information into the shares is prevented	The model is not tested under attacks
Singh et al. [23] (2018)	Basis matrices and error diffusion	No pixel expansion Alignment of shares not required for reconstruction No need of explicit codebook for construction	Construction of the secret shares is performed in three steps adding to computational complexity
Huang et al. [33] (2021)	Basis matrices and error correction mechanism of QR codes	The approach is tested with a wide range of attacks	The secret code and all the shares are of same version. Though it is the underlying working principle of the method, when the number of shares increase more memory and transmission bandwidth will be required
Chen et al. [34] (2022)	*(n*,*n)* threshold and error correction mechanism	Facilitates sharing of WeChat Mini Program codes	Robustness of the approach is demonstrated with attacks

**Table 2:** Experimental results for QR code sharing and reconstruction.

Original QR Code	Secret Share *S*_1_	Secret Share *S*_2_	Secret Share *S*_3_	Reconstructed QR Code	PSNR in dB	SSIM	Readability
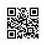	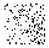	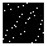	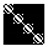	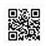	32.3639	0.9373	Yes
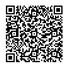	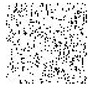	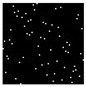	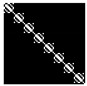	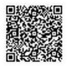	32.0880	0.9313	Yes
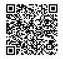	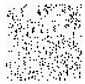	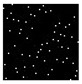	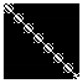	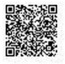	32.2787	0.9310	Yes
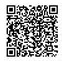	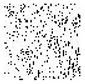	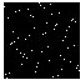	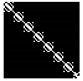	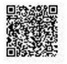	32.2555	0.9298	Yes
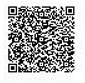	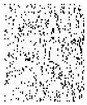	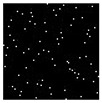	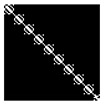	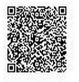	32.3889	0.9297	Yes
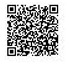	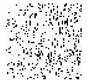	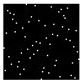	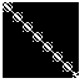	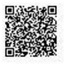	32.2646	0.9265	Yes
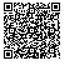	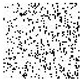	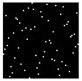	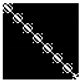	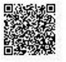	31.5443	0.9201	Yes
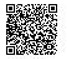	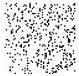	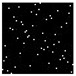	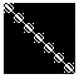	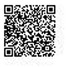	31.6609	0.9128	Yes
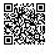	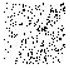	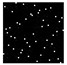	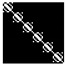	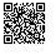	32.1306	0.9110	Yes
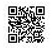	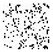	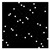	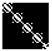	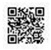	31.3103	0.9091	Yes

**Table 3:** Comparisons of significant attributes.

Methods	Recovery of Secret Image	Shadow Size with Respect to Secret Image Size	Pixel Expansion	Pre-Encryption & Decryption	Complexity
Naor & Shamir [18]	Lossy	1	No	No	*O*(*klog*_2_*k*)
Thien and Lin [19] (lossy)	Lossy	1/*k*	No	Yes	*O*(*k*^3^)
Thien and Lin [19] (lossless)	Lossless	1/*k*	Yes	Yes	*O*(*k*^3^)
Yang et al. [20]	Lossless	1	No	No	High
Ding et al. [21]	Lossless	1	No	No	*O*(*k*^3^)
Zhou et al. [22]	Lossless	1/1 − *k*	No	Yes	*O*(*k*^3^)
Zhou et al. [22](without permutation)	Lossless	1/1 − *k*	No	No	*O*(*k*^3^)
Singh et al. [23]	Lossless	1	No	No	*O*(*n*)
Huang et al. [33]	Lossless	1	No	No	*O*(*n*)
Chen et al. [34]	Lossless	1	No	No	*O*(*n*)
**Proposed Method**	**Lossless**	**<=1**	**No**	**No**	***O*(*n*)**

**Table 4:** Comparison of execution times.

Method	Sharing Time (s)	Recovery Time (s)
Naor & Shamir [18]	7.721	7.831
Thien and Lin [19] (lossy)	1.792	2.764
Ding et al. [21]	138.219	10.585
Zhou et al. [22]	1.732	2.424
Zhou et al. [22] (mod 257)	2.714	3.205
Singh et al. [23]	1.3219	0.0615
**Proposed Method**	**0.2436**	**0.0910**

**Table 5:** No. of combinations for construction of shares.

Size of Secret	*m*	No. of Combinations		
		Singh et al. [23]	Proposed Method	
			Minimum	Maximum
		$2^{3m^{2}}$	$2^{2m+3}$	$2^{3m^{2}+2m}$
29 × 29	29	2^2523^	2^61^	2^2581^
33 × 33	33	2^3267^	2^69^	2^3333^
41 × 41	41	2^5043^	2^85^	2^5125^
45 × 45	45	2^6075^	2^93^	2^6165^
53 × 53	53	2^8427^	2^109^	2^8533^
57 × 57	57	2^9747^	2^117^	2^9861^
61 × 61	61	2^11,163^	2^125^	2^11,285^
77 × 77	77	2^17,787^	2^157^	2^17,941^

**Table 6:** Number of iterations of Arnold Transform to unscramble the secret.

Size of Secret	*d*	No. of Iterations of Arnold Transform 3(2*^d^*^−2^)
29 × 29	29	3 × 2^27^
33 × 33	33	3 × 2^31^
41 × 41	41	3 × 2^39^
45 × 45	45	3 × 2^43^
53 × 53	53	3 × 2^51^
57 × 57	57	3 × 2^55^
61 × 61	61	3 × 2^59^
77 × 77	77	3 × 2^75^

**Table 7:** Total number of computations to recover the secret.

Secret Size	Singh et al. [23]	No. of Computations to Recover the Secret [Proposed System]	
		Minimum	Maximum
29 × 29	2^2524^	3 × 2^92^	3 × 2^2612^
33 × 33	2^3268^	3 × 2^104^	3 × 2^3368^
41 × 41	2^5044^	3 × 2^128^	3 × 2^5168^
45 × 45	2^6076^	3 × 2^140^	3 × 2^6212^
53 × 53	2^8428^	3 × 2^164^	3 × 2^8588^
57 × 57	2^9748^	3 × 2^176^	3 × 2^9920^
61 × 61	2^11,164^	3 × 2^188^	3 × 2^11,348^
77 × 77	2^17,788^	3 × 2^236^	3 × 2^18,020^

**Table 8:** No. of computations for LR to HR conversion.

Secret Size	No. of Computations
29 × 29	1.14 × 10^8^
33 × 33	1.48 × 10^8^
41 × 41	2.28 × 10^8^
45 × 45	2.74 × 10^8^
53 × 53	3.81 × 10^8^
57 × 57	4.40 × 10^8^
61 × 61	5.04 × 10^8^
77 × 77	8.03 × 10^8^

## Data Availability

Not applicable.
